# Opioid system in L-DOPA-induced dyskinesia

**DOI:** 10.1186/s40035-017-0071-y

**Published:** 2017-01-17

**Authors:** Jing Pan, Huaibin Cai

**Affiliations:** Transgenics Section, Laboratory of Neurogenetics, National Institute on Aging, National Institutes of Health, Building 35, Room 1A112, MSC 3707, 35 Convent Drive, Bethesda, MD 20892-3707 USA

## Abstract

L-3, 4-Dihydroxyphenylalanine (L-DOPA)-induced dyskinesia (LID) is a major clinical complication in the treatment of Parkinson’s disease (PD). This debilitating side effect likely reflects aberrant compensatory responses for a combination of dopaminergic neuron denervation and repeated L-DOPA administration. Abnormal endogenous opioid signal transduction pathways in basal ganglia have been well documented in LID. Opioid receptors have been targeted to alleviate the dyskinesia. However, the exact role of this altered opioid activity is remains under active investigation. In the present review, we discuss the current understanding of opioid signal transduction in the basal ganglia and how the malfunction of opioid signaling contributes to the pathophysiology of LID. Further study of the opioid system in LID may lead to new therapeutic targets and improved treatment of PD patients.

## Background

Parkinson’s disease (PD) is the most common degenerative movement disorder clinically manifested with resting tremor, bradykinesia, rigidity and posture instability, resulting from impairments of dopamine transmission in the basal ganglia [1]. Pathologically, PD is marked by the substantial degeneration of dopamine-producing neurons in the substantia nigra pars compacta (SNc) and the presence of intracellular protein aggregates named Lewy bodies and neurites [2]. PD medications are mostly targeted at symptom relief. Since its initial prescription in 1960s, dopamine precursor L-DOPA remains the most effective drug for treating the movement abnormalities [3]. However, from the very beginning, it has been noticed that repeated administration of L-DOPA can induce motor fluctuations as well as impulsive control disorders [4]. Involuntary movements, also called L-DOPA induced dyskinesia (LID), is the most disturbing motor fluctuation. LID can be disabling and interfere with daily living. On average, about half of the patients develop LID after treated with L-DOPA for five years [5]. Although intensive studies have been carried out to understand the underlying molecular, cellular, and circuit mechanisms of LID, there still lack agents that can effectively ameliorate LID. Therefore, it remains critical to develop novel therapeutic strategies to reduce LID and improve the treatment and life quality of PD patients.

Denervation of dopaminergic neurons and repeated L-DOPA treatment might act together to cause LID [6]. The development of LID reflects multiplex compensatory reactions of nervous system in response to innate dopamine transmission deficiency and excessive supply of L-DOPA as discussed comprehensively in a recent review [7]. Here we paid special attention to the opioid receptor-mediated neurotransmission. Endogenous opioid peptides are dopamine co-transmitters that modulate various neural transmissions in basal ganglia. Alterations of opioid peptide expression and opioid receptor-mediated intracellular signal transduction have been reported in PD patients and animal models that develop dyskinesia [8]. Targeting opioid signaling in the basal ganglia may provide an additional avenue for the treatment of LID in PD.

## Endogenous opioid peptides and receptors

There are three families of endogenous opioid peptides and three families of opioid receptors, comprising the so-called endogenous opioid system in the brain [9]. Endogenous opioid peptides consist of β-endorphin, enkephalins, and dynorphins, which are derived from precursor proteins encoded by pre-proopiomelanocortin (POMC), preproenkephalin (PENK), and pre-prodynorphin (PDYN), respectively [10]. Each precursor undergoes complex post-translational modifications and proteolytic cleavage, giving rise to multiple active peptides [11, 12]. For instance, PENK is the precursor for two extended forms of Methionine (Met)-enkephalin and a single form of Leucine (Leu)-enkephalin. Opioid receptors can be divided into μ, δ, and κ three families, encoded by OPRM, OPRD, and OPRK genes [4, 13, 14]. While they are all G protein–coupled receptors, their extracellular loops are less conserved and responsible for the differential binding affinities to different opioid peptides [4, 13, 15–17]. All three subtypes of opioid receptors are coupling with the downstream G_i_ or G_o_-mediated inhibitory intracellular signaling transduction pathways [18]. Furthermore, endogenous opioid peptides and receptors exhibit uneven distribution in different brain regions and cell types, and can act either pre- or post-synaptically. Therefore, the opioid system exerts a variety of modulatory roles in multiple neural processes, including pain sensation, reward and drug addiction, as well as seizure and PD [11].

## Functional organization of basal ganglia

**Fig. 1:**
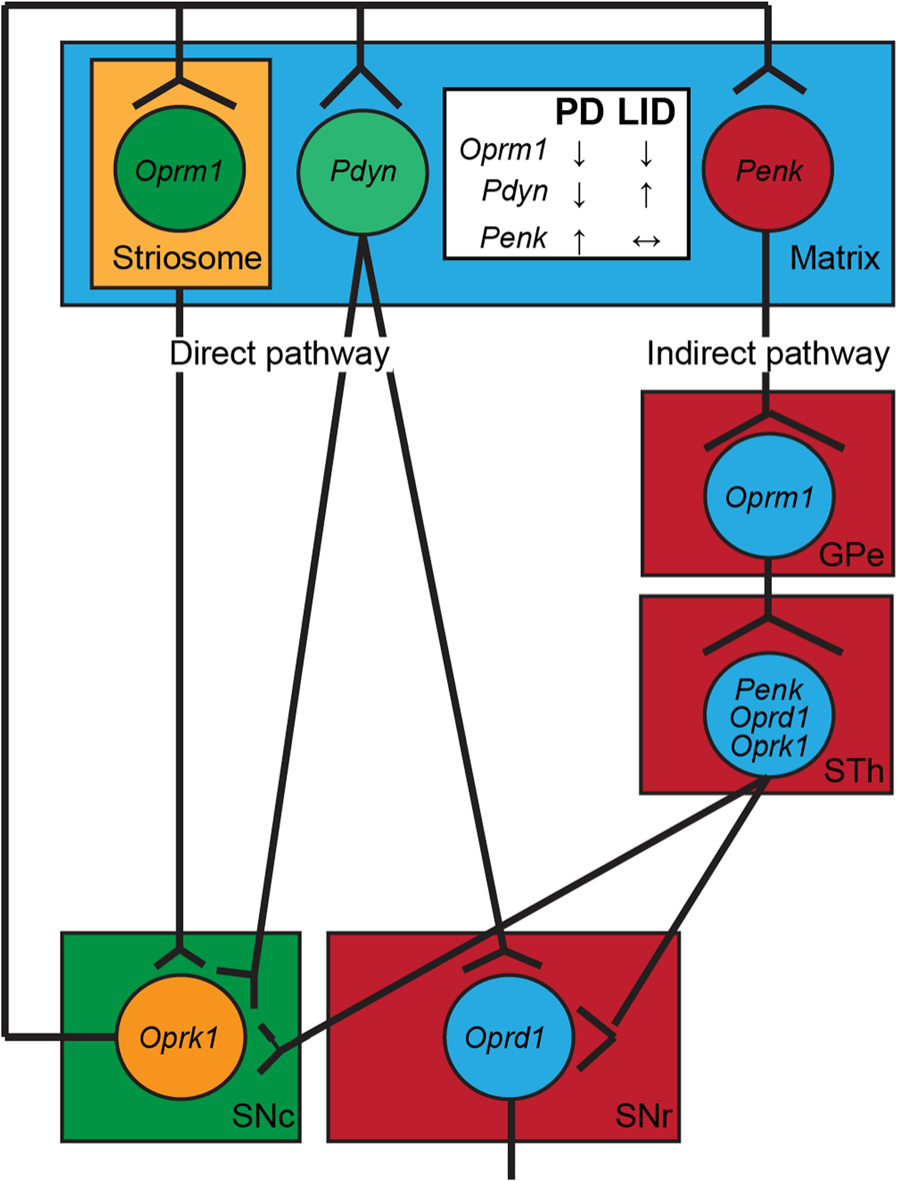
Endogenous opioid peptide and receptor gene expression and alteration in basal ganglia under PD and LID conditions. A sketch of basal ganglia local circuit map shows characteristic expression patterns of endogenous opioid peptide and receptor genes in different sub-regions. The inset highlights signature alterations of opioid peptide and receptor expression in the dorsal striatum under PD and LID conditions. Oprm1: μ opioid receptor; Oprd1: δ opioid receptor; Oprk1: κ opioid receptor; Penk: preproenkephalin; Pdyn: pre-prodynorphin; GPe: globus pallidus external segment; STh: subthalamic nucleus; SNc: substantia nigra pars compacta; SNr: substantia nigra pars reticulata

Increasing evidence suggests that well-coordinated direct and indirect striatal outputs in the basal ganglia are essential for the proper motor control [19]. On the other hand, a disruption of the coordination may cause parkinsonian as well as dyskinetic states [20]. The dopamine receptor D1 (DRD1)-expressing striatal projection neurons (dSPNs) form the direct pathway and project to the globus pallidus internal segment (GPi) as well as the substantia nigra pars reticulata (SNr) and SNc, while the dopamine receptor D2 (DRD2)-expressing SPNs (iSPNs) make up the indirect pathway and innervate the globus pallidus external segment (GPe) and the subthalamic nucleus (STh), where the GPe and STh neurons target their axons to the SNr (Fig. 1) [19, 20]. The direct pathway is used to be regarded as a promoter of movement, in opposite to the indirect pathway that inhibits the motor activity [19]. Along this line of thinking, PD weakens the direct pathway and potentiates the indirect pathway, resulting in impairments of movement [19, 21]. However, the recent live imaging studies of SPNs in free moving rodents argue against this binary “to go or not to go” model, and suggest a concerted activity of direct and indirect pathway SPNs is essential for the proper motor control in both moving and resting states [22–24]. In addition, the latest neuron tracing studies reveal a much more complicated and diverse connectivity of SNPs, as well as the midbrain dopaminergic neurons (Fig. 1) [25–27]. Further dissecting the local circuits and functional modalities of basal ganglia neurons will likely redraw the wiring of neural network that underlies the critical motor control of vertebrates.

## Opioid system in basal ganglia

High levels of endogenous opioid peptides and opioid receptors are present in the basal ganglia [11]. Because the wide distribution of opioid peptides and receptors in the brain, it is difficult to determine whether the sources of these peptides and receptors are inside or outside of basal ganglia based on studying the protein expression using western blot and immunostaining. By contrast, the distribution of mRNAs determined by in situ hybridization serves a better indicator to the patterns of opioid peptide and receptor expression in basal ganglia (Fig. 1). The endogenous opioid peptide precursor PENK mRNAs are predominantly expressed by the iSNPs, whereas PDYN mRNAs are more abundant in the dSNPs [8, 28]. By contrast, the expression of POMC mRNAs is restricted to hypothalamus. *Penk* but not *Pdyn* mRNAs can also be detected in neuronal subpopulations in subthalamic nucleus and zona incerta in rodent brains (Allen Brain Atlas), regions targeted for deep brain stimulation (DBS) in treatment of PD and LID [29]. All three types of opioid receptors express in the striatum, but display distinct distribution patterns. The μ opioid receptors (OPRM1) are selectively expressed by the SPNs located in the striosome compartments in the rodent brains [30]. Correlatively, Oprm1 mRNAs also show a similar patchy distribution pattern in rodent dorsal striatum (Allen). In addition, Oprm1 mRNAs appear at ventral striatum and GPe (Allen Brain Atlas). δ-opioid receptors (Oprd1) mRNA-expressing cells sparsely distribute at dorsal striatum, GPe, GPi, and SNr, while more cells express Oprd1 in STh and ZI. κ-opioid receptors (Oprk1) mRNAs are mainly detected in cells distributed at ventral striatum and STh. Of interest, Oprk1 mRNAs are more enriched in SNc and ventral tegmental area (VTA) compared to other opioid peptides and receptors (Allen Brain Atlas). These distinct sub-regional localizations of opioid peptides and receptors underlie the diverse and complex functions of endogenous opioid system in the basal ganglia [10].

## Alterations of striatal opioid peptide and receptor expression in LID

The expression levels of striatal PENK mRNAs correlate with the severity of LID, suggesting a causal role of increased opioid transmission in the development of LID [17, 31]. However, the pathophysiological impact of these alterations remains debatable [32]. Dopamine depletion as occurred in the parkinsonian states leads to increased expression of PENK mRNA in the iSPNs and decreased expression of PDYN mRNAs in the dSPNs (Fig. 1) [33, 34]. At the peak of LID, the expression of PDYN mRNAs increases, while the expression of PENK does not change (Fig. 1) [8, 33–35]. The expression of PDYN mRNAs and peptides also elevates in STh neurons in LID [34, 36]. However, such alterations of opioid peptide expression may not cause LID, but rather reflect an adaptive response compensating for the prolonged L-DOPA treatment [37, 38].

Correlated with the alterations of opioid peptide expression, opioid receptor levels also change in PD and LID (Fig. 1) [39]. Dopamine depletion causes an overall reduction of opioid receptor bindings, while LID leads to a further reduction [34]. OPRM1 binding levels are decreased in both the caudate and putamen of PD patients under chronic L-DOPA administration [40, 41]. OPRM1 levels are also suppressed in putamen and GPi of monkey LID models [42]. On the other hand, OPRM1 expression is increased in the premotor and motor cortex of rat LID models [43]. Therefore, it is important to investigate the differential alterations of opioid peptide and receptor expression in different brain regions in PD and LID.

## Opioid signaling in the pathogenesis of LID

While OPRM1 cell surface presentation is decreased in LID, OPRM1-mediated signal transduction is enhanced in the striatum and GPi of LID models [44], suggesting increased sensitivity of OPRM1 in the dyskinetic states. Consistent with this notion, OPRM1 antagonists cyprodine and ADL5510 effectively reduce dyskinesia but preserve the anti-parkinsonian efficacy of L-DOPA in the monkey models [38, 45]. Moreover, a combination of both OPRM1 and OPRD1 antagonists appear more effective in reducing LID in animal models [46]. The anti-LID efficacy of OPRM1 and OPRD1 antagonists remains to be validated in PD patients, especially considering an earlier failed attempt with the non-selective opioid receptor antagonist naloxone [47, 48]. In addition, the diverse expression patterns of opioid receptors in different brain regions and their differential responses in the dyskinesia states may demand regional application of either receptor subtype-selective agonists or antagonists to effectively alleviate LID.

The underlying molecular mechanisms of LID remain under intensive investigation [49]. Dysregulation of cAMP and ERK signaling pathways has been reported in PD and LID models [50]. Opioid receptors relay opioid stimulation through G_i_ or G_o_-mediated inhibitory intracellular signal transduction pathways [18], which may suppress G_olf_ or G_s_-induced cAMP signaling in cells. For example, pharmacological activation of OPRM1 and OPRD1 blocks dopamine DRD1- or adenosine A2A-receptor-induced activation of adenylyl cyclase and reduces the production of cAMP in SPNs [51]. A reduction of cAMP levels may suppress protein kinase A (PKA) activity and leads to an aberrant activation of extracellular signal-regulated kinases (ERK1/2) in the generation of LID [52, 53]. ERK activation may play a causal role in the development of LID, as inhibition of ERK activity alleviates dyskinesia phenotypes [54]. Opioid receptor antagonists may achieve the anti-dyskinesia effects through negatively regulating ERK1/2 signaling [55]. Further studies, however, will be required to improve current understanding of the complex interplays between various signaling pathways critical in the formation of abnormal synaptic transmission and plasticity in LID.

## Conclusions

LID represents an erratic adaptive response to dopamine denervation and repeated L-DOPA treatment. Although selective opioid receptor antagonists can effectively alleviate LID in animal models, the underlying molecular, cellular and circuit mechanisms remain to be characterized. The latest advance of next generation sequencing, gene editing, opt genetics, and live imaging technologies may greatly facilitate the inquiry of opioid signaling in the basal ganglia under normal and PD-like conditions. Modulation of opioid receptor activity may provide an effective means to reduce LID and optimize the dopamine-replacement therapy.
